# Centriolar Protein POC5 Regulates Human Adipogenesis and Cellular Senescence: Insights From a Novel Metabolic Ciliopathy

**DOI:** 10.1096/fj.202601789R

**Published:** 2026-07-27

**Authors:** Valeria Pistorio, Camille Vatier, Émilie Capel, Carine Beaupère, Martine Auclair, Virginie Steunou, Romain Morichon, Michael Joubert, Corinne Vigouroux, Jérémie Gautheron, Isabelle Jéru

**Affiliations:** ^1^ Centre de Recherche Saint‐Antoine (CRSA) Inserm, Sorbonne Université Paris France; ^2^ Foundation for Innovation in Cardiometabolism and Nutrition (IHU‐ICAN) Paris France; ^3^ Department of Endocrinology, Diabetology and Reproductive Endocrinology Assistance Publique‐Hôpitaux de Paris, Saint‐Antoine University Hospital, National Reference Center for Rare Diseases of Insulin Secretion and Insulin Sensitivity (PRISIS), Endo‐ERN Center for Rare Endocrine Diseases Paris France; ^4^ Cytometry and Imagery Platform Saint‐Antoine (CISA) Inserm UMS30 Lumic Paris France; ^5^ Endocrinology and Diabetes Department CHU Côte de Nacre Caen France; ^6^ Department of Medical Genetics, DMU BioGeMH, Pitié‐Salpêtrière Hospital Sorbonne Université, AP‐HP Paris France

**Keywords:** centriolar protein, ciliogenesis, insulin resistance, lipodystrophy, POC5, premature senescence

## Abstract

Centrosomes and primary cilia regulate cellular processes, including microtubule organization and lineage‐specific differentiation. POC5, a core component of the centriolar inner scaffold, has been linked to syndromic ciliopathies, yet its role in adipose biology remains unclear. This study investigates the impact of POC5 deficiency on ciliary organization, cellular senescence, adipogenesis, and insulin signaling. To this end, we analyzed primary dermal fibroblasts from a patient carrying a novel homozygous p.(Gln206Ter) POC5 variant and performed mechanistic evaluations in human adipose stem cells (ASCs) with CRISPR‐Cas9‐mediated POC5 knockout. Centriolar architecture was examined using Ultrastructure Expansion Microscopy (U‐ExM), and cellular phenotypes were assessed through proliferation, senescence, and signaling analyses. POC5‐deficient fibroblasts showed marked disruption of centriolar architecture, including absent or abnormal primary cilia and supernumerary centrioles. These defects were associated with a 35% decrease in proliferation and a premature senescence, evidenced by increased SA‐β‐gal activity and upregulation of p‐p53, p16, and p21. Moreover, insulin signaling was impaired, with reduced phosphorylation of IRβ, AKT, and ERK1/2. These phenotypes were recapitulated in POC5‐KO ASCs, which additionally exhibited a near complete block of adipogenic differentiation, associated with downregulation of PPARγ, C/EBPα, and SREBP1c. Overall, POC5 deficiency promotes insulin resistance and premature senescence, and impaired adipogenesis. These findings identify POC5‐related disease as a centrosomal metabolic disorder and highlight the importance of centriolar integrity in systemic energy homeostasis, supporting the need for metabolic monitoring in individuals with POC5 pathogenic variants.

## Graphical abstract

The identification of a patient carrying a novel homozygous p.(Gln206Ter) *POC5* variant revealed a metabolic phenotype associated with *POC5* deficiency. *POC5* deficiency disrupts centriolar architecture and ciliary organization, leading to impaired proliferation, premature cellular senescence, and reduced insulin signaling. In human adipose stem cells, *POC5* loss blocks adipogenic differentiation and decreases key adipogenic regulators, including PPARγ, C/EBPα, and SREBP1c. These findings identify POC5‐related disease as a centrosomal metabolic ciliopathy affecting adipogenesis and cellular homeostasis.

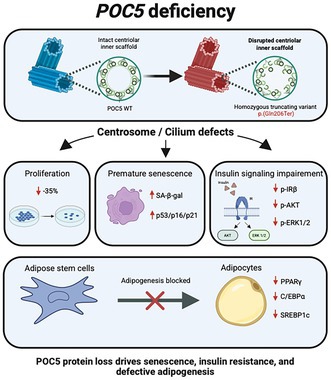

## 1 Introduction

Centrosomal proteins and the primary cilium coordinate diverse cellular functions, from microtubule organization to growth factor signaling and differentiation [1]. Among them, POC5 (Proteome Of Centriole protein 5), a WD40 repeat‐containing centriolar protein, localizes to the distal end of the centriole, where it contributes to centriole elongation and structural stability [2, 3, 4]. Through its role in assembling the distal centriole scaffold, POC5 ensures proper ciliogenesis and maintenance of centrosome integrity, processes that are essential for cellular signaling and tissue homeostasis [2]. Structurally, POC5 has been characterized as a conserved centrin‐binding protein that forms part of a helical inner scaffold, along with POC1B, Centrin‐2, and FAM161A, crucial for maintaining microtubule triplet (MTT) cohesion and geometry [3].

Initially implicated in an autosomal recessive form of retinitis pigmentosa [5], biallelic loss‐of‐function variants in *POC5* were more recently shown to cause a syndromic retinal, endocrine, and neuromuscular ciliopathy, expanding the phenotypic spectrum of POC5‐related disease [6]. The syndrome is characterized by multi‐organ involvement, including rod‐cone retinal dystrophy (RCD), diabetes mellitus with severe insulin resistance, partial lipodystrophy (LD), kidney disease, and muscle cramps. *POC5* variants have also been associated with adolescent idiopathic scoliosis (AIS) [7, 8, 9] and common polymorphisms in *POC5* have been linked to complex traits such as type 2 diabetes (T2D) in Genome‐Wide Association Studies (GWAS) [10]. These findings indicate that POC5 plays broader roles in human physiology than previously appreciated. Nevertheless, potential metabolic consequences of *POC5* deficiency, particularly its impact on adipose tissue function and systemic energy homeostasis, have not yet been investigated at the cellular mechanistic level.

POC5 belongs to the same protein family as POC1A, which when biallelically inactivated causes SOFT syndrome, a primordial dwarfism with partial LD and severe insulin/IGF‐1 resistance [11, 12, 13]. Building on these observations, we hypothesized that POC5, like POC1A, may regulate insulin sensitivity and adipocyte biology [11, 14]. The intersection between centrosomal structure and metabolic disorders is further exemplified by the discovery of genetic variants in *ALMS1*, which cause Alström syndrome (AS) characterized by childhood obesity, severe insulin resistance, RCD, and dilated cardiomyopathy with adipose tissue dysfunction [15, 16, 17]. Similarly, pathogenic variants in *PCNT* (Pericentrin) cause Microcephalic Osteodysplastic Primordial Dwarfism type II (MOPDII) and are associated with severe insulin resistance, diabetes, and adipogenesis defects [18].

Here, we report a patient with a previously‐unreported homozygous nonsense *POC5* variant presenting with retinal dystrophy, scoliosis, and a metabolic phenotype comprising severe insulin resistance, dyslipidemia, and hepatic steatosis. Using patient‐derived fibroblasts and CRISPR‐Cas9 knockout ASCs, we demonstrate that *POC5* deficiency leads to defective insulin signaling, enhanced cellular senescence, and abrogated adipocyte differentiation. Together, these findings extend the clinical and functional landscape of *POC5* deficiency and establish POC5 as a central regulator linking centrosomal integrity to metabolic homeostasis, defining a newly emerging class of centrosomal metabolic disorders.

## 2 Materials and Methods

### 2.1 Study Approval

Written informed patient consent was obtained for the genetic study and publication of data. The study was approved by the CPP Ile de France 5 research ethics board (DC 2009‐963, Paris, France).

### 2.2 Genetic Analyses

Genomic DNA was extracted from peripheral blood leukocytes. using standard procedures. Whole Exome Sequencing (WES) was performed by IntegraGen SA (Evry, France) using the Twist Human Core Exome Enrichment System (Twist Bioscience, OR, USA) and IntegraGen Custom, followed by paired‐end 75 bases massively parallel sequencing on Illumina HiSeq4000. Data analysis was performed using Sirius software (IntegraGen SA). *POC5* variant was described based on the longest isoform (NM_001099271.2) using Alamut 2.11 (Sophia Genetics, Lausanne, Switzerland) and the Human Genome Variation Society guidelines. LD‐related genes were screened using a targeted gene panel as previously described [19, 20, 21]. Sanger sequencing. (Big Dye Terminator v3.1, Thermo Fisher Scientific, MS, USA) was used for variant validation on a 3500xL Dx device.

### 2.3 Clinical, Biological and Imagery Investigations

Clinical data were obtained during routine follow‐up. Metabolic investigations included measurements of fasting plasma glucose, insulin, glycated hemoglobin (HbA1c), lipid profile, hepatic transaminases, leptin, and adiponectin. Body fat percentage and trunk‐to‐limb fat ratio were measured by Dual energy X‐rays absorptiometry (DEXA).

### 2.4 Cellular Modeling of the Disease in Fibroblasts

Primary fibroblast cultures from the patient were established after skin biopsy. Cultured fibroblasts from control women were studied at similar passages as control as previously described [14]. Cells were grown in DMEM low glucose with pyruvate (Thermo Fisher Scientific) supplemented with 10% fetal bovine serum (Dutscher, Issy‐les‐Moulineaux, France), 1% penicillin/streptomycin, and 2 mM glutamine (Invitrogen, CA, USA).

### 2.5 Cellular Modeling of the Disease in Human ASC

CRISPR/Cas‐9 mediated deletion of *POC5* was performed in ASCs obtained from controls with a guide RNA (gRNA) targeting *POC5* exon 1 (sense nucleotide sequence 5′‐GTGCAAAATGACTCCAGTCG‐3′) using a previously described method [19, 22, 23]. Cells transfected with a Cas9/scramble gRNA plasmid were used as controls. Adipocyte differentiation of ASCs was performed as previously described [20, 21]. Cells were studied before exposure to the adipogenic medium and/or after 20 days of differentiation.

### 2.6 Western Blot Studies

Protein expression studies were performed on whole cell extracts (Table 1 for antibodies). Protein detection and semi‐quantitative analysis of Western Blot vs. tubulin were performed in triplicate using the iBright CL1500 imaging system (Invitrogen). The effect of insulin on signaling intermediates was assessed on cells maintained in fetal calf serum‐free medium for 24 h, then stimulated by 100 nM insulin for 8 min.

**TABLE 1:** Key resources table.

Reagent type or resource	Designation	Source and reference	Identifiers	Additional information
Adipose stem cells	ASC	Pr. Fève lab at CRSA, Paris	N/A	Female, from subcutaneous abdominal adipose tissue
Antibody	Anti‐adiponectin	Thermo Fisher Scientific	Cat# MA1‐054	WB (1:1000)
Antibody	Anti‐AKT	Cell Signaling Technology	Cat# 9272	WB (1:1000)
Antibody	Anti‐C/EPBα	Protein Tech	Cat# 18311‐1‐1P	WB (1:1000)
Antibody	Anti‐ERK	Cell Signaling Technology	Cat# 9102	WB (1:1000)
Antibody	Anti‐FAS	Cell Signaling Technology	Cat# 3180	WB (1:1000)
Antibody	Anti‐IRβ	Cell Signaling Technology	Cat# 3025	WB (1:1000)
Antibody	Anti‐P16	Protein Tech	Cat# 10883‐1‐AP	WB (1:1000)
Antibody	Anti‐P21	Protein Tech	Cat# 10355‐1‐AP	WB (1:1000)
Antibody	Anti‐P53	Abcam	Cat# ab1101	WB (1:1000)
Antibody	Anti‐P‐AKT	Cell Signaling Technology	Cat# 9271	WB (1:1000)
Antibody	Anti‐perilipin	Abcam	Cat# ab3526	WB (1:1000)
Antibody	Anti‐P‐ERK	Cell Signaling Technology	Cat# 9101	WB (1:1000)
Antibody	Anti‐POC5	Novus	Cat# NBP2‐76534	IF (1:1000)
Antibody	Anti‐P‐P53	Abcam	Cat# ab38497	WB (1:1000)
Antibody	Anti‐PPARγ	Protein Tech	Cat# 16643‐1‐AP	WB (1:1000)
Antibody	Anti‐SREBP‐1	Santa Cruz Biotechnology	Cat# sc‐366	WB (1:1000)
Antibody	Anti‐tubulin	Sigma‐Aldrich	Cat# T5168	WB (1:10 000)
Antibody	Anti‐P‐Tyr	Santa Cruz Biotechnology	Cat# sc‐7020	WB (1:500)
Antibody	Anti‐γ‐tubulin	Sigma	Cat# T6557	IF (1:100)
Antibody	Anti‐ARL13B	Proteintech	Cat# 17711‐1‐AP	IF (1:100)
Antibody	Anti‐rabbit‐HRP	Cell Signaling Technology	Cat# 7074	WB (1:3000)
Antibody	Anti‐mouse‐HRP	Cell Signaling Technology	Cat# 7076	WB (1:3000)
Antibody	Anti‐rabbit‐AlexaFluor 488	Invitrogen	Cat# A21206	IF (1:100)
Lectins	Wheat Germ Agglutinin (WGA) Alexa Fluor 633	Invitrogen	Cat# W21404	IF (1:200)
Antibody	Anti‐mouse‐AlexaFluor 546	Invitrogen	Cat# A10036	IF (1:100)
Recombinant DNA reagent (plasmid)	lentiCRISPR v2	Addgene	Cat# 52961	A gift from Zhang lab
Software algorithm	Prism	Graphpad Software	N/A	

*Note:* This table lists the key antibodies and reagents used in this study, including references, supplier information, and experimental conditions.

### 2.7 Intracellular Triglyceride Quantification

Quantification of intracellular lipid content was performed as previously described [20]. Intracellular TGs were extracted from differentiated ASC using hexane/isopropyl alcohol (3:2). Cells were washed and incubated with hexane/isopropyl alcohol (3:2, vol/vol) using 500 μL per well in 6‐well culture plates, in a shaker (80 rpm/min) at room temperature for 60 min. The content of each well was then transferred into a glass tube for nitrogen evaporation of the organic solvent. After evaporation, lipids were resuspended in isopropyl alcohol and transferred into duplicate 96‐well plates for analysis after drying. Triglycerides were measured using the Infinity Triglyceride kit (Thermo Fischer Scientific) according to manufacturer's instructions. The absorbance of each well was measured using a Tecan microplate reader (TECAN, Männedorf, Switzerland) and converted to concentration based on a standard curve. Results were normalized to the cell protein content.

### 2.8 Fluorescence and Immunofluorescence Microscopy Studies

Cells grown on glass coverslips were fixed in methanol at −20°C and stained with DAPI for nuclear visualization. To induce ciliogenesis, cells reaching 80%–90% confluence were cultured in serum‐free media for 48 h. Centrioles and cilia were identified using antibodies against γ‐tubulin and ARL13B, respectively. Adipocyte lipid storage was quantified via Oil Red‐O staining as previously described [24]. Cell proliferation was assessed by BrdU incorporation, while senescence‐associated β‐galactosidase activity was measured through X‐Gal staining [25]. Images were acquired using Olympus IX83 and Olympus FV3000 confocal microscope, and cellSens software (Evident, Rungis, France).

### 2.9 Ultrastructure Expansion Microscopy (U‐ExM)

Fibroblasts were grown on 12 mm coverslips, then fixed for 10 min in iced‐cold methanol. Fixed WT‐ and *POC5*‐KO cells were expanded as previously described [26]. Briefly, cells were incubated in anchoring solution (1.4% paraformaldehyde (PFA), 2% acrylamide (AA) in PBS) for 5 h at 37°C. Cells were then rinsed twice in 1× PBS and incubated 10 min on ice, on a parafilm‐lined ice‐cooled humidified chamber, atop a 35 μL monomere solution: 19.3% sodium acrylate, 10% AA, 0.1% N,N′‐methylenbisacrylamide in PBS, polymerized by adding 2.5 μL of TEMED 50% and 2.5 μL of APS (0.1 g/mL). The chamber was then transferred for 1 h at 37°C to induce complete gelation. Cells embedded in gel were then transferred in a 6 well‐plate into denaturation buffer (200 mM SDS, 200 mM NaCl, 50 mM Tris H_2_O pH 9) then placed at 95°C for 90 min in an Eppendorf tube filled with denaturation buffer. Gels were then hydrated 3 times for 30 min minimum in 100 mL ddH_2_O at RT, no agitation. The next day, gels were shrunk back in 1× PBS, cut into 1 cm^2^ pieces and transferred into a 24‐well plate. Gels were incubated for 30 min in PBS 1% BSA at 37°C with gentle agitation, then in primary antibodies for 3 h at 37°C with gentle agitation. Gels were rinsed 3× 10 min in PBS tween 0.1%, then incubated in secondary antibodies. Following antibodies labeling, gels were hydrated 3× in ddH_2_O, then mounted onto poly‐L‐Lysine coated glass μ‐Slide chambers (Ibidi GmbH, Gräfelfing, Germany). For CRISPR‐control and CRISPR *POC5*‐KO, POC5, and tubulin staining, samples were counterstained with Wheat Germ Agglutinin (WGA) Alexa Fluor 633 (Invitrogen, W21404; 1:200) to label cell membranes. *Z*‐stack acquisitions were made on an Olympus FluoView 3000 IX83 confocal laser scanning microscope using an oil UPLSAPO 60× objective. Expansion factor of 5.5 was estimated by comparing nucleus size before and after expansion. Representative 3D videos of uExM cilia were obtained on Imaris software V10.2 using standard deconvolution parameters and a median filter in *x*, *y*, *z*.

### 2.10 Statistical Analyses

Data from at least three independent experiments are expressed as means SEM (or SD when specified). Statistical significance (*p* < 0.05) was assessed using one‐way or two‐way ANOVA followed by Bonferroni's post hoc test, as appropriate, via GraphPad Prism 8 (CA, USA). For cilia analysis, contingency tables were analyzed using Fisher's exact test on pooled cell counts.

## 3 Results

### 3.1 Identification of a *POC5* Homozygous Variant in a Patient With a Complex Insulin Resistant Syndrome

The proband was a young adult woman from Algeria born from consanguineous parents. She was considered asymptomatic until the age of 18 years, when she complained of abdominal pain revealing hepatomegaly and liver steatosis, along with elevated levels of aspartate aminotransferase, alanine aminotransferase, and gamma glutamyl transpeptidase. She displayed dysmorphic features including microcephaly, tall forehead, triangular face with pointed chin and prominent nose, sparse hair, ptosis, and brachydactyly. She had a high‐pitched voice and complained of visual impairment. Her height and weight were normal (163 cm, 48 kg, body‐mass index 18.1 kg/m^2^) but she presented with a truncal distribution of fat with a relative lipoatrophy of the four limbs. She was simultaneously diagnosed with metabolic dysfunction‐associated steatotic liver disease with cirrhosis complicated by portal hypertension and esophageal varices, high blood pressure, and diabetes (HbA1c 13.8%), which required very high doses of insulin therapy (6 U/kg/d). Insulin resistance was also characterized by marked *acanthosis nigricans* (upper and lower limb folds, neck) and polycystic ovary syndrome with severe hirsutism and oligoamenorrhea occurring rapidly after puberty. Her body fat percentage was in the lower range (19.7%) with an increased trunk‐to‐limb fat ratio [1, 25]. Serum leptin and adiponectin were low (3.8 and 0.72 mg/L, respectively). Triglycerides were very high (27.1 mmol/L), with low HDL‐cholesterol (0.86 mmol/L). Ophthalmological examination revealed retinitis pigmentosa without diabetic retinopathy. Proteinuria was diagnosed with normal glomerular filtration rate. Clinical cardiovascular assessment was normal as well as echocardiography.

**FIGURE 1:**
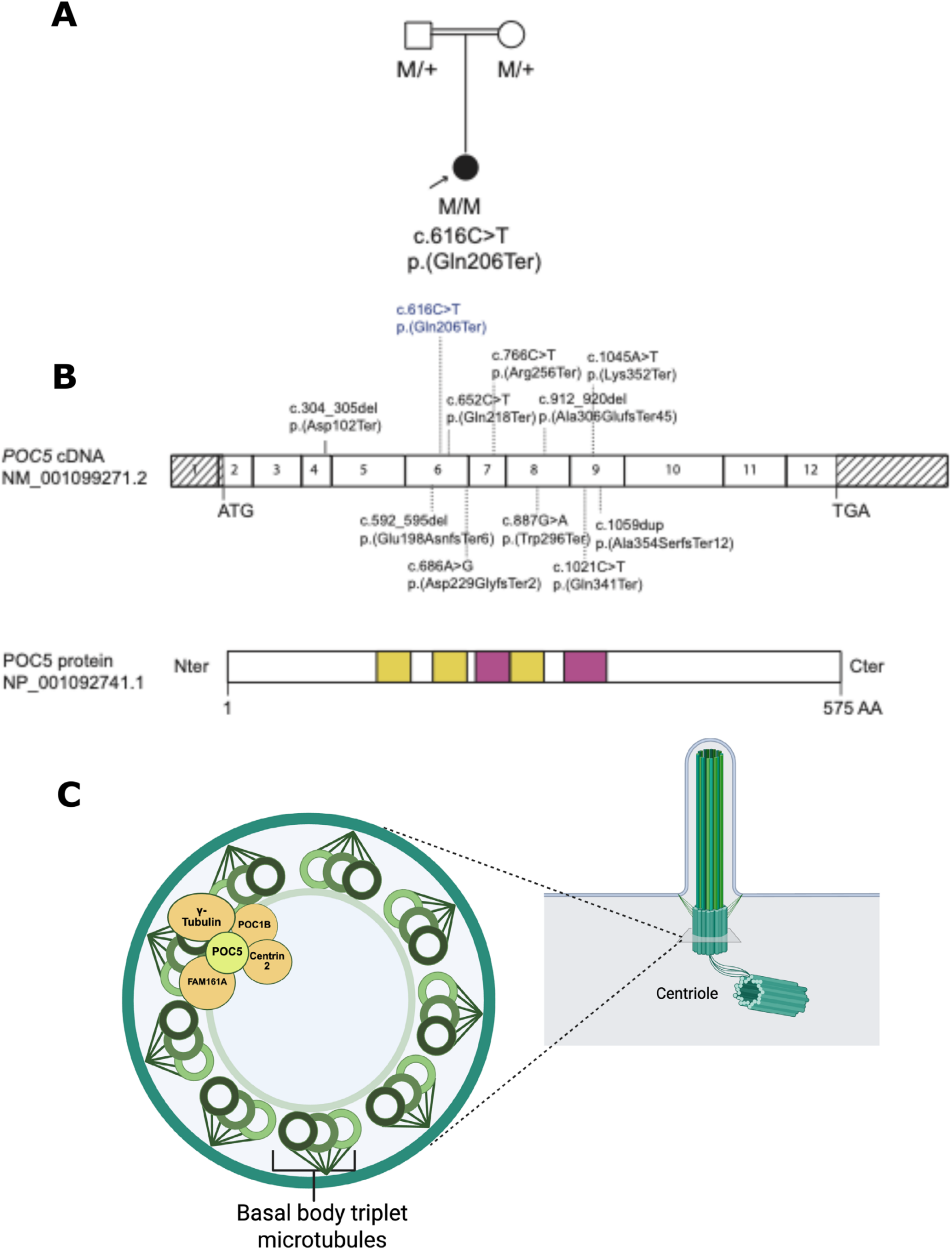
Identification of a *POC5* homozygous null variant in a patient with a complex insulin resistant syndrome. (A) Genealogical tree and segregation analysis for the *POC5* variant. The arrow indicates the proband. +, normal allele; M, mutant allele. (B) Top panel: Schematic representation of *POC5* transcript sequence (NM_001099271.2) displaying in blue the location of the variant identified herein, and in black the variants identified in previous reports. Bottom panel: Schematic representation of POC5 protein sequence comprising 575 amino acids. The prediction of protein domain organization was based on UniProt database (protein reference: Q8NA72). The predicted Centrin Binding Repeats (CBR) are indicated in orange, the putative coiled‐coil regions are depicted in pink. (C) POC5 localizes along the inner wall of the centriole lumen together with POC1B, Centrin‐2, FAM161A, and γ‐tubulin, forming an inner scaffold associated with the microtubule triplets. Created with BioRender.com.

WES was carried out in trio with the index case and her two unaffected parents (Figure 1A). The disease of the index case was not explained by variants in classical genes known to be involved in insulin resistant syndromes, as assessed by the analysis of a panel of genes used in routine genetic diagnosis. WES led to the identification of a homozygous nonsense variant in exon 6 of *POC5*: c.616C>T; p.(Gln206Ter) (NM_001099271.2) (Figure 1B), which was present in the heterozygous state in both parents (Figure 1A). Genotypes were confirmed by Sanger sequencing. This nonsense variant was identified only once in the heterozygous state in the gnomAD database (v4.1.0) reporting variants from the general population (minor allele frequency: 10^−6^). No alternative molecular etiology was retained to explain the disease phenotype when considering the different inheritance modes compatible with the familial history (*de novo*, compound heterozygous, and homozygous variants).

The clinical manifestations of the patient investigated herein are presented in Table 2, as compared to previously reported cases of patients carrying biallelic loss‐of‐function variants [5, 6]. This synthetic view shows that *POC5* deficiency emerges as a pleiotropic syndrome where metabolic hallmarks are nearly constant, including severe insulin resistance or diabetes and liver steatosis. Of particular note is the high prevalence of partial lipodystrophy, observed in over half of the patients (7/13), a clinical feature that is generally uncommon in classical ciliopathies but appears central to this specific centrosomal disorder.

**TABLE 2:** Summary of clinical manifestations reported in patients with biallelic *POC5* pathogenic variants.

Clinical manifestation	Weisz Hubshman et al. [5] (*n* = 1)	Vulto‐van Silfhout et al. [6] (*n* = 12^a^)	Present study (*n* = 1)	Total number of patients (*n* = 13)
Facial dysmorphism^b^	0/1	6/12	1/1	7/13
Integumentary (Sparse hair/Alopecia)	0/1	7/12	1/1	8/13
Growth (Short stature/Microcephaly)	1/1	12/12	1/1	13/13
Skeletal (Scoliosis/Kyphosis)	0/1	2/12	1/1	3/13
Ocular (Rod‐cone dystrophy/Retinitis pigmentosa)	1/1	11/12	1/1	12/13
Metabolic (Diabetes/Insulin resistance)	0/1	10/12	1/1	11/13
Adipose tissue (Partial lipodystrophy)	0/1	6/12	1/1	7/13
Hepatic (Steatosis/NAFLD/Cirrhosis)	0/1	10/12	1/1	11/13
Renal (Glomerulonephritis/Insufficiency)	1/1	7/12	1/1	8/13
Neuromuscular (Muscle cramps/Elevated CK)	1/1	10/12	0/1	10/13

^a^ The cohort in Vulto‐van Silfhout et al. [6] includes the patient previously reported by Weisz Hubshman et al. [5] as Participant 1 (P1).

^b^ Facial dysmorphism includes features such as wide nasal base, mandibular prognathia, large ears, or triangular face.

### 3.2 Loss of POC5 Protein Expression and Defects in Centrosome Organization and Ciliogenesis in *POC5* ‐Deficient Fibroblasts

**FIGURE 2:**
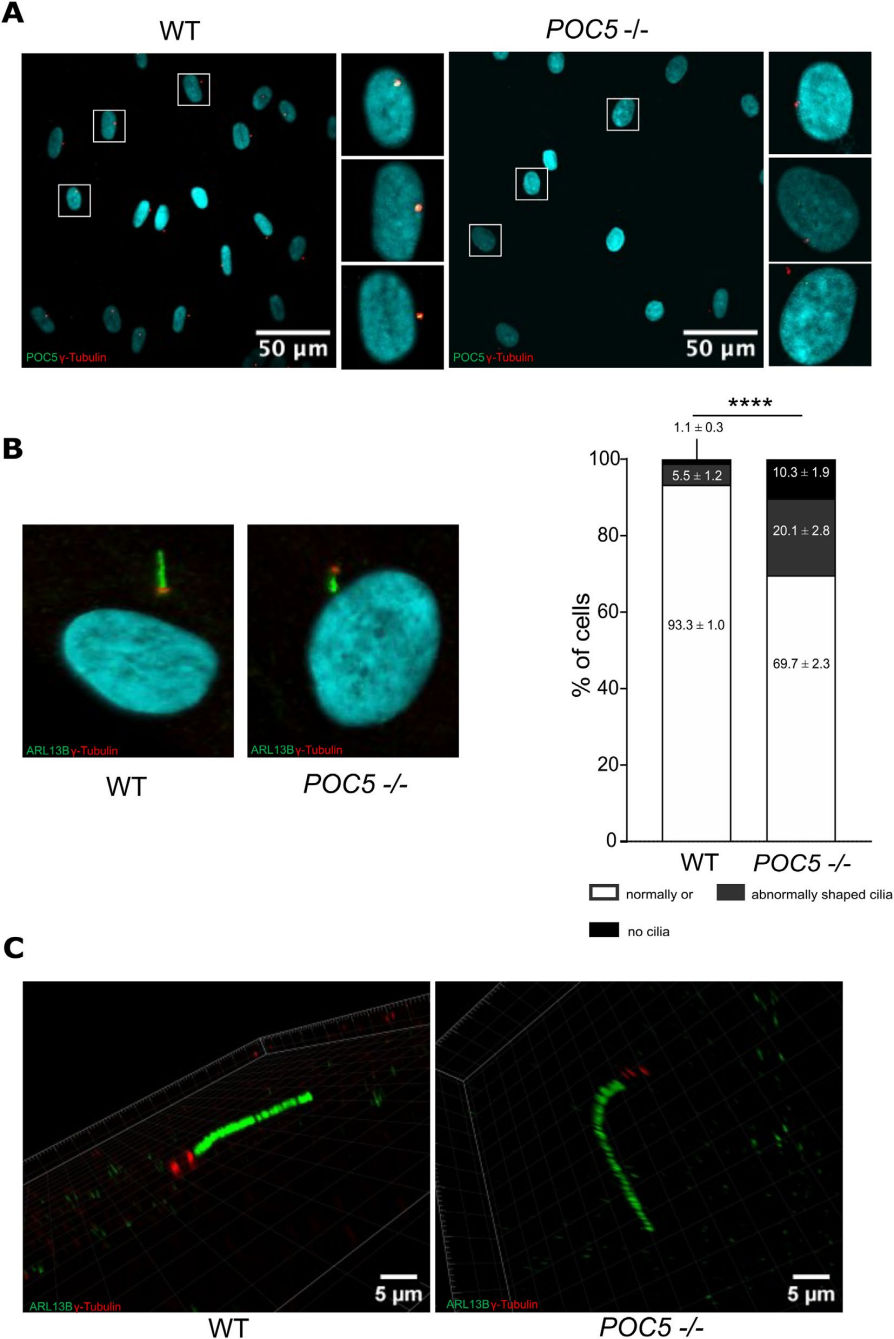
Fibroblasts from the patient harboring a pathogenic variant show a lack of POC5 protein expression and defects in cilia and basal body organization. (A) IF images of cycling control (WT) and *POC5* ^−/−^ cells stained against the indicated proteins (green) and the centrosomal marker γ‐tubulin (red). Scale bar: 50 μm. Representative photographs are shown, with magnification of cells depicted by rectangles. (B) Immunocytological features of cilia and centrosome/basal body organization in fibroblasts from controls and the patient. Cell nuclei are stained in blue with DAPI. Centrioles are revealed in red by anti‐γ‐tubulin staining, and cilia in green by anti‐ARL13B staining. The percentage of cells with normal cilia (white boxes), abnormally shaped cilia (gray boxes), and of cells without cilia (black boxes) was evaluated on a total number of 597 control and 198 patient's cells and expressed as means ± SD. Ciliary phenotypes in WT and *POC5* ^−/−^ cells were quantified and pooled from three independent experiments. Normal versus abnormal and absent cilia were compared using Fisher's exact test. ****p < 0.0001 vs. control. (C) Representative frames from high‐resolution videos of ciliary architecture and centrosomes, captured using Ultrastructure Expansion Microscopy (U‐ExM) and provided in the S1 and S2, in fibroblasts from controls and the patient. Centrioles are revealed in red by anti‐γ‐tubulin staining, and cilia in green by anti‐ARL13B staining. Patient fibroblast shows multiple centrioles and disrupted axonemal organization, when compared to more regular ciliary architecture in control fibroblasts. Scale bar: 5 μm.

The *POC5* gene is ubiquitously expressed. Taking advantage of this broad expression pattern, we established primary dermal fibroblast cultures from a skin biopsy of the proband. *POC5* encodes a 575‐amino acid protein (63.4 kDa) that localizes to the distal end of the centriole, where it contributes to centriole formation and primary cilium assembly (Figure 1C) [2]. The biallelic nonsense variant identified in the patient (c.616C>T; p.(Gln206Ter)) is located in exon 6 of a 12‐exon gene and is predicted to trigger nonsense‐mediated mRNA decay, as previously shown for *POC5* loss‐of‐function alleles [6]. To assess the consequences of this variant at the protein level, immunofluorescence analysis was performed (Figure 2A). In control fibroblasts (WT), POC5 localized as a discrete signal at the distal end of centrioles stained with γ‐tubulin (Figure 2A). In contrast, no detectable POC5 signal was observed in patient‐derived fibroblasts, confirming loss of protein expression (Figure 2A). Given the established role of POC5 in distal centriole formation and ciliogenesis, we next examined centrosome and cilium organization (Figure 2B,C). Immunofluorescence microscopy revealed marked structural abnormalities in patient fibroblasts compared with controls. Cells exhibited a high frequency of short, abnormally curved, or truncated primary cilia, and in a substantial fraction of cells, cilia were entirely absent (Figure 2B,C). Using Ultrastructure Expansion Microscopy (U‐ExM), we obtained high‐resolution views of ciliary architecture. As shown in the Supporting Information Videos S1 and S2, patient‐derived fibroblasts exhibit markedly disrupted axonemal organization, including shortened, swollen, or misoriented cilia, further corroborating the profound ciliogenesis defects seen in conventional immunofluorescence (Supporting Information Videos S1 and S2). Moreover, centrosomal fragmentation and supernumerary centrioles were frequently observed, indicative of a disturbed centriole duplication and basal‐body architecture (Figure 2C). Collectively, these findings demonstrate that biallelic *POC5* variants cause severe defects in centrosome integrity and ciliogenesis, establishing a cellular basis for the broad phenotypic spectrum, including retinal, skeletal, and metabolic manifestations.

### 3.3 Accelerated Senescence, Impaired Proliferative Capacity, and Insulin Resistance in *POC5* ‐Deficient Fibroblasts

**FIGURE 3:**
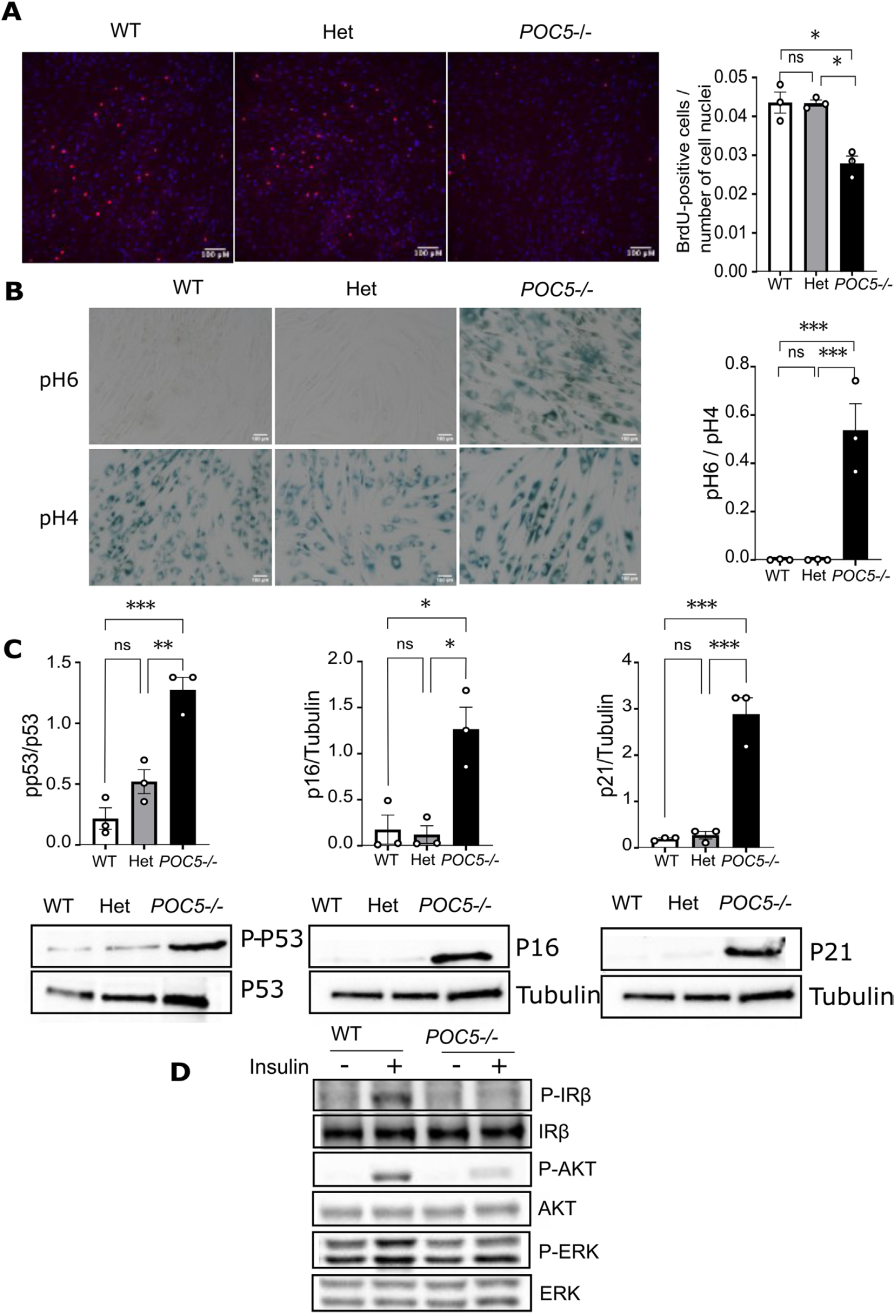
Fibroblasts from the patient with a homozygous *POC5* variant show impaired proliferation capacity, increased senescence markers and impaired insulin sensitivity. (A) Cellular proliferation was assessed by the capacity of fibroblasts to incorporate BrdU. Cell nuclei stained by anti‐BrdU antibodies were identified by immunofluorescence microscopy (red staining) and counted relative to total nuclei (blue DAPI staining). Quantification of BrdU fluorescence was normalized to DAPI staining and expressed as means ± SD of 3 independent experiments. Scale bar: 100 μm. (B) Senescence‐activated‐β‐galactosidase activity of cells was assessed by the ratio of X‐gal staining at pH 6 to nonspecific staining at pH 4. Representative images and quantitative measurements from 3 independent experiments are shown (means ± SEM). Scale bar: 100 μm. (C) The protein expression of the cell cycle arrest markers phosphorylated‐p53 (P‐P53) as compared to total P53, and P16 and P21 normalized to tubulin, was evaluated by Western blotting in fibroblasts from controls and the patient. For P16 and P21, the same tubulin loading control immunoblot was used because the membrane was stripped and reprobed for P16, P21, and tubulin. Representative blots (from 3 independent experiments) and quantitative measurements (means ± SD) are shown. (D) Insulin activation of signaling intermediates was evaluated by Western blotting in fibroblasts from controls, heterozygote (Het) and the patient. The total protein expression of the signaling intermediates insulin receptor β‐subunit (IRβ), protein kinase B (AKT) and extracellular‐regulated kinase (ERK) 1/2, and of their insulin‐activated forms (P‐: Phosphorylated proteins) were evaluated using antibodies listed in Table 1. Insulin‐stimulated phosphorylation of IRβ was assessed using antibody directed against phospho‐tyrosine residue. Tubulin is an index of the cellular protein level. Blots are representative of four independent experiments. Western blot quantifications are available in Figure S1. Statistical analysis was performed using one‐way or two‐way ANOVA followed by Bonferroni's post hoc test. *p < 0.05**p < 0.01, ***p < 0.001 vs. control.

Dysfunction of the primary cilium has been associated with accelerated cellular and tissue aging, which can contribute to growth retardation, defective adipose tissue homeostasis, and resistance to insulin [14]. To determine whether the patient's biallelic *POC5* variants induce similar defects, we evaluated the replicative capacity and senescence profile of patient fibroblasts compared to heterozygous parental fibroblasts and age‐matched controls. Cell proliferation, assessed by BrdU incorporation, was significantly reduced in *POC5*‐deficient fibroblasts, with a 35% decrease relative to controls (*p* < 0.05) and no reduction in heterozygous cells (Figure 3A). In line with impaired proliferation, senescence‐associated β‐galactosidase (SA‐β‐gal) activity, assessed under pH 6 conditions (which selectively detects senescent cells), was markedly elevated in *POC5*‐deficient fibroblasts (Figure 3B). At pH 6, all patient fibroblasts stained positive compared to negligible activity in heterozygous and control fibroblasts, which remained negative under these conditions. At pH 4 (positive control), all fibroblast populations showed robust β‐galactosidase activity, confirming assay validity. This shift indicates a true senescence‐specific increase in β‐gal activity in patient cells rather than baseline enzymatic differences. Consistent with these findings, Western blot analyses demonstrated robust upregulation of canonical cell cycle arrest and senescence markers (Figure 3C). Phosphorylated P53 (P‐P53) levels were approximately doubled, while P16 (INK4a) and P21 (CIP1) were increased at least fourfold relative to control and heterozygous fibroblasts. These results indicate that *POC5* deficiency triggers a premature senescence program in patient fibroblasts, likely contributing to impaired tissue growth and mirroring the senescence phenotype previously described in *POC5*‐null cells (e.g., cell cycle arrest in G1 or extended S phase) [2, 7].

In addition to the senescent phenotype, patient fibroblasts displayed marked insulin resistance (Figures 3D and S1). Following insulin stimulation, phosphorylation of the insulin receptor β subunit (IRβ), AKT, and ERK1/2 was significantly reduced compared to controls, with decreases ranging from 41% to 66% depending on the signaling intermediate examined. These combined defects indicate that *POC5* deficiency not only induces a premature senescence program but also disrupts proximal insulin signaling in fibroblasts, a dual impairment that likely contributes to the metabolic phenotype observed in the patient.

### 3.4 CRISPR‐Mediated *POC5* Knockout Disrupts Ciliogenesis and Recapitulates the Senescent and Insulin‐Resistant Phenotype

To determine whether loss of *POC5* alone is sufficient to reproduce the structural and functional defects observed in patient‐derived fibroblasts, we generated *POC5* knockout (*POC5*‐KO) human ASC using CRISPR‐Cas9 with guide targeting exon 1 of *POC5*. Non‐targeting guide‐transduced ASC (CRISPR Ctrl) and wild‐type (WT) ASC served as controls. To assess whether the ciliary abnormalities observed in patient fibroblasts were directly attributable to *POC5* loss, we next examined primary cilium organization in *POC5*‐KO ASC. As in *POC5*‐deficient fibroblasts, immunofluorescence analysis revealed a marked reduction in the percentage of ciliated cells, along with the presence of short or abnormally shaped primary cilia (Figure S2). Moreover, centrosomal abnormalities, including centriole fragmentation and the presence of supernumerary centrioles, were frequently observed, confirming that POC5 loss alone is sufficient to disrupt centriole integrity and ciliogenesis (Figure S2). These findings demonstrate that POC5 plays an essential role in maintaining centrosome architecture and primary cilium assembly across distinct human cell types.

**FIGURE 4:**
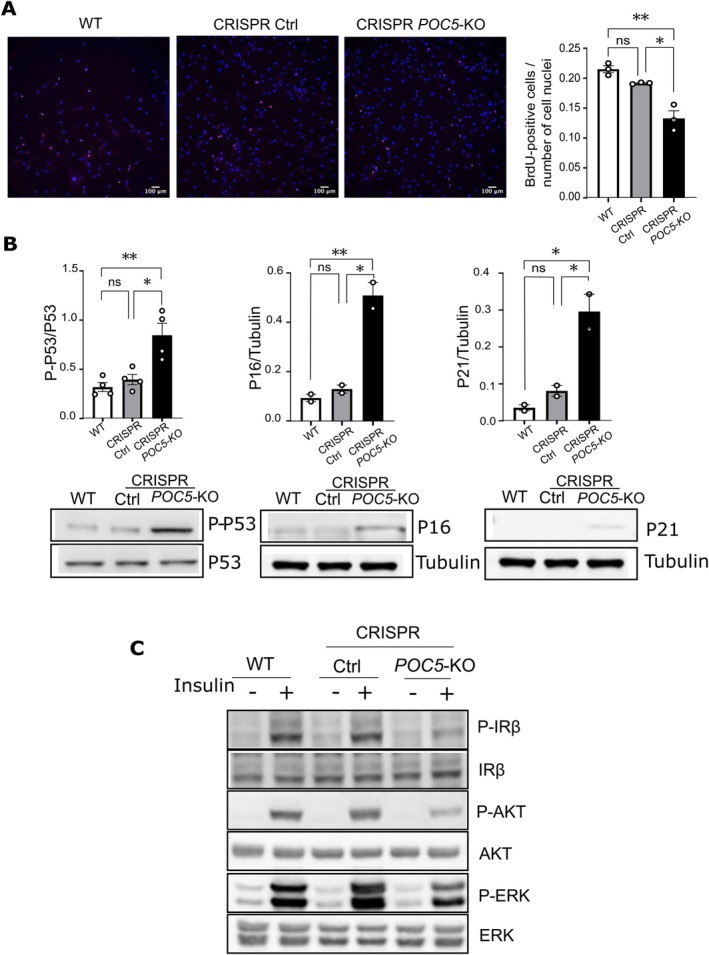
CRISPR‐Cas9‐mediated deletion of *POC5* in ASCs recapitulates impaired proliferation and increased senescence and resistance to insulin. Data were obtained in control ASCs (WT), ASCs transfected with a Cas9/scramble gRNA plasmid (CRISPR‐Ctrl), or ASCs submitted to a CRISPR‐Cas9‐mediated *POC5*‐knockout (CRISPR *POC5*‐KO), cultured in a maintenance medium (non‐differentiated ASCs). Experiments were conducted as described in fibroblasts. (A) Cellular proliferation was assessed by the capacity of ASCs to incorporate BrdU. Cell nuclei stained by anti‐BrdU antibodies were identified by immunofluorescence microscopy (red staining) and counted relative to total nuclei (blue DAPI staining). Quantification of BrdU fluorescence was normalized to DAPI staining and expressed as means ± SD of three independent experiments. Scale bar: 100 μm. (B) The protein expression of the cell cycle arrest markers phospho‐p53 (P‐P53) as compared to total P53, and P16 and P21 normalized to tubulin, was evaluated by Western blotting. For P16 and P21, the same tubulin loading control immunoblot was used because the membrane was stripped and reprobed for P16, P21, and tubulin. Representative blots (from 3 independent experiments) and quantitative measurements (means ± SD) are shown. (C) Insulin activation of signaling intermediates was evaluated by Western blotting in control ASCs (WT), ASCs transfected with a Cas9/scramble gRNA plasmid (CRISPR‐Ctrl), or ASCs submitted to a CRISPR‐Cas9‐mediated *POC5*‐knockout (CRISPR *POC5*‐KO). The total protein expression of the signaling intermediates insulin receptor β‐subunit (IRβ), protein kinase B (AKT) and extracellular‐regulated kinase (ERK) 1/2, and of their insulin‐activated forms (P‐: Phosphorylated proteins) were evaluated using antibodies listed in Table 1. Insulin‐mediated phosphorylation of IRβ was assessed using antibody directed against phospho‐tyrosine residue. Tubulin is an index of the cellular protein level. Blots are representative of three independent experiments. Western blot quantifications are available in Figure S3. Statistical analysis was performed using one‐way or two‐way ANOVA followed by Bonferroni's post hoc test. *p < 0.05, **p < 0.01 vs. WT and control.

Proliferative capacity was markedly impaired in *POC5*‐KO ASC, as shown by a 38% reduction in BrdU incorporation compared with both CRISPR Ctrl (*p* < 0.05) and wild‐type ASC (*p* < 0.01) (Figure 4A). Consistent with this, WB analysis revealed robust induction of senescence‐associated pathways, with P‐P53, P21, and p16 levels each elevated by approximately 2.6 to 8‐fold relative to controls (at least *p* < 0.05 for all comparisons) (Figure 4B). In addition to the senescent phenotype, *POC5*‐KO ASC demonstrate a decreased insulin response (Figures 4C and S3). Following insulin stimulation, activation of IRβ, AKT, and ERK1/2 by phosphorylation was reduced by 37% to 51% compared with CRISPR Ctrl and WT ASC. These results indicate that *POC5* deficiency intrinsically impairs insulin‐mediated activation of proximal signaling intermediates. Collectively, these data confirm that *POC5* loss drives premature senescence and blunted insulin signaling, recapitulating the dual cellular hallmarks observed in patient‐derived fibroblasts.

### 3.5 *POC5* Deletion Abolishes Adipocyte Differentiation and Triggers Insulin Resistance

**FIGURE 5:**
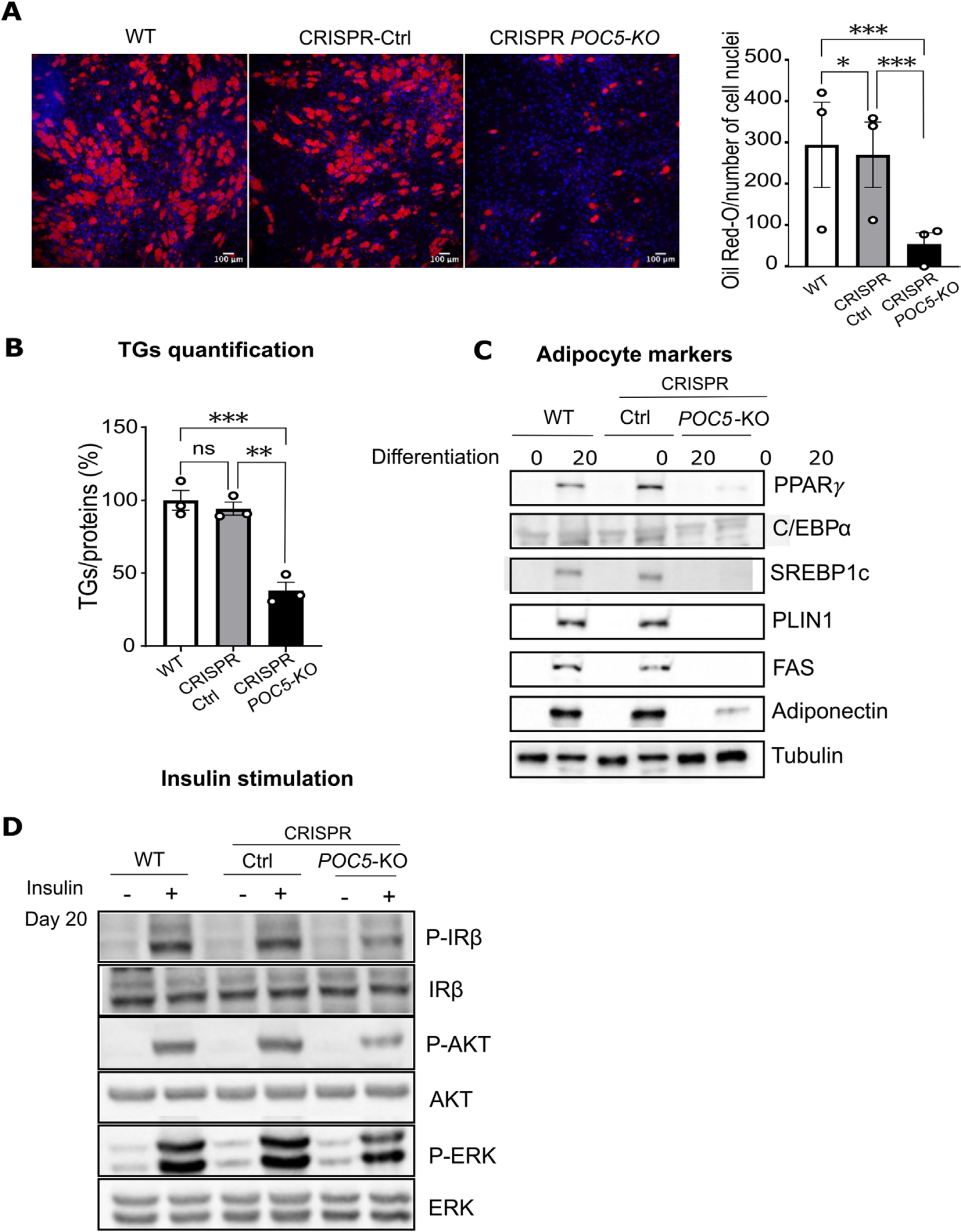
CRISPR‐Cas9‐mediated deletion of *POC5* impairs adipocyte differentiation of human adipose stem cells. Data were obtained in control ASCs (WT), ASCs transfected with a Cas9/scramble gRNA plasmid (CRISPR Ctrl), or ASCs submitted to a CRISPR‐Cas9‐mediated *POC5*‐knockout (CRISPR *POC5*‐KO), cultured in a maintenance medium (non‐differentiated cells, day 0) or in an adipogenic medium for 20 days as indicated (day 20). (A) Oil Red‐O red staining of intracellular lipids was evaluated at day 20 of adipocyte differentiation by fluorescence microscopy. Cell nuclei are stained in blue by DAPI. Representative images are shown. Quantification of Oil Red‐O fluorescence was normalized to DAPI staining and expressed as means ± SD of 3 independent experiments. (B) Intracellular triglyceride content was measured during in vitro adipocyte differentiation. Triglycerides were measured in control ASCs (WT), ASCs transfected with a Cas9/scramble gRNA plasmid (CRISPR Ctrl), or ASCs submitted to a CRISPR‐Cas9‐mediated *POC5*‐knockout (CRISPR *POC5*‐KO), after 20 days of adipocyte differentiation. Measurements are representative of three independent experiments. (C) Protein expression of adipogenic factors and mature adipocyte markers as assessed by Western blotting in ASCs studied at day 0 and day 20 of adipocyte differentiation. PPARγ, peroxisome proliferator‐activated receptor gamma; C/EBPα, CCAAT/enhancer binding protein alpha; SREBP1c, sterol regulatory element‐binding protein 1c; PLIN1, perilipin‐1; FAS, fatty acid synthase. Tubulin is used as a loading control. Images are representative of 3 independent experiments. (D) Activation of insulin signaling in adipocytes after 20 days of adipocyte differentiation was evaluated by Western blotting in control ASCs (WT), ASCs transfected with a Cas9/scramble gRNA plasmid (CRISPR Ctrl), or ASCs submitted to a CRISPR‐Cas9‐mediated *POC5*‐knockout (CRISPR *POC5*‐KO). The total protein expression of the signaling intermediates insulin receptor β‐subunit (IRβ), protein kinase B (AKT) and extracellular‐regulated kinase (ERK) 1/2, and of their insulin‐activated forms (P‐: Phosphorylated proteins) were evaluated using antibodies listed in Table 1. Insulin‐mediated phosphorylation of IRβ was assessed using antibody directed against phospho‐tyrosine residue. Tubulin is an index of the cellular protein level. Western Blots are representative of 3 independent experiments. Western blot quantifications are available in Figures S4 and S5. Statistical analysis was performed using one‐way or two‐way ANOVA followed by Bonferroni's post hoc test. *p < 0.05, **p < 0.01, ***p < 0.001 vs. WT and control.

WT ASC and CRISPR Ctrl ASC efficiently differentiated into mature adipocytes within 20 days (D20). Differentiation was confirmed by progressive accumulation of refractive lipid droplets visible by optical microscopy, robust Oil Red O staining, and a marked increase in intracellular triglyceride content (Figure 5A). In contrast, *POC5*‐KO ASC exhibited a near‐complete failure to differentiate. At D20, these cells showed an ~80% reduction in Oil Red O staining (*p* < 0.001) and an ~62% decrease in triglyceride content (at least *p* < 0.01) relative to WT and control cells (Figure 5B). To explore the molecular basis of this defect, we examined the expression of key transcriptional regulators and markers of adipogenesis and mature adipocytes by Western Blot (Figures 5C and S4). Compared with WT and CRISPR Ctrl ASC, *POC5*‐KO cells displayed a sharp reduction in the expression of adipogenic transcription factors, including peroxisome proliferator‐activated receptor gamma (PPARγ), CCAAT/enhancer‐binding protein‐alpha (C/EBPα), and sterol regulatory element‐binding protein‐1c (SREBP1c) at D20. Mature adipocyte markers such as fatty acid synthase (FAS), perilipin (PLIN1), and adiponectin were also strongly decreased, indicating a global block in the differentiation program (Figures 5C and S4).

We next assessed the impact of *POC5* loss on insulin signaling during adipogenesis (Figures 5D and S5). In WT and CRISPR Ctrl adipocytes at D20, insulin stimulation induced strong phosphorylation of the insulin receptor β subunit (IRβ), AKT, and ERK1/2, consistent with robust mitogenic and metabolic pathway activation. In contrast, *POC5*‐KO cells at D20 were markedly resistant to insulin, as evidenced by a near‐absence or strong reduction in phosphorylation of these intermediates. Strikingly, this defect was already present at the preadipocyte stage (D0), with a 41% to 55% reduction in insulin‐stimulated phosphorylation of IRβ, AKT, and ERK compared with CRISPR Ctrl ASC, indicating that the insulin resistance observed at D20 is not solely a consequence of impaired differentiation but, at least in part, an intrinsic feature of *POC5* deficiency. These findings collectively demonstrate that *POC5* loss disrupts insulin signaling and abrogates the adipogenic program, contributing to the LD phenotype and systemic insulin resistance observed in *POC5*‐deficient individuals.

## 4 Discussion

This study expands the clinical and cellular spectrum of *POC5* deficiency, underlining the crucial role of this centrosomal protein in a complex syndrome with metabolic dysfunction characterized by primary insulin resistance, premature cellular senescence, and abrogated adipocyte differentiation.

Our results confirm and extend recent observations showing that biallelic loss‐of‐function variants in *POC5* cause a multiorgan syndromic ciliopathy across 12 families [6]. This syndrome includes retinal, renal, muscular, and severe endocrine abnormalities; metabolic manifestations comprised insulin‐resistant diabetes and signs of partial LD in six participants. The patient in the current study presented with partial LD, severe insulin‐resistant diabetes, *acanthosis nigricans*, low serum adiponectin levels, hyperandrogenism, and hypertriglyceridemia, supporting the prominence of the metabolic involvement. The absence of homozygous *POC5* null variants in the gnomAD database (v4.1.0) supports the deleterious nature of complete loss of function.

At the cellular level, *POC5* loss resulted in centrosome and primary cilium disorganization (including abnormally curved or absent cilia), activation of p‐p53‐p16‐p21 pathways, and reduced proliferation, consistent with accelerated senescence. This senescence program, observed in patient fibroblasts and *POC5*‐KO ASCs, likely contributes to impaired adipocyte differentiation from mesenchymal progenitors. Beyond cellular aging, the structural integrity of the primary cilium is intrinsically required for adipogenic commitment, acting as a specialized antenna that senses and integrates extrinsic signals [27, 28]. The primary cilium is essential during the early stages of differentiation to sensitize mesenchymal progenitors to pro‐adipogenic hormones, specifically by facilitating the recruitment and localized activation of the IGF‐1 receptor and AKT at the ciliary base [29, 30]. Moreover, recent studies have identified the ciliary localization of the omega‐3 fatty acid receptor FFAR4/GPR120 as a crucial driver of adipogenesis through the rapid production of ciliary cAMP and activation of the EPAC signaling cascade [31, 32]. The profound ciliary disorganization observed in *POC5* deficiency likely abrogates these early sensory functions, thereby establishing a direct link between ciliary dysfunction and the adipogenic failure observed in our patient. Furthermore, defects in proximal insulin signaling observed prior to differentiation indicate that *POC5* loss intrinsically induces insulin resistance. The convergence of insulin resistance, cellular senescence, and blocked adipogenesis defines a maladaptive mitotic and postmitotic state underlying LD‐like features and systemic metabolic dysfunction. These findings echo mechanisms described for *POC1A*‐related SOFT syndrome, suggesting that POC5 and POC1A define a subgroup of centriolar disorders intersecting with systemic metabolism [11, 12].

Orthologues of *POC5* are conserved across eukaryotes, reflecting its ancient role in centriole architecture [33]. POC5 is a component of the distal centriole, physically interacting with Centrin‐2 and Centrin‐3 [2, 3], and is required for assembly of the distal half of centrioles [2]. Together, POC5 and POC1A exemplify a group of centrosomal metabolic disorders, in which disruption of distal centriole components leads to insulin resistance through defects in ciliary signaling and adipocyte differentiation. Other centrosomal proteins, including ALMS1 (Alström syndrome) [15] and PCNT (MOPDII) [18], produce overlapping syndromes characterized by insulin resistance, dyslipidemia, and multi‐organ involvement with adipose tissue dysfunction. ALMS1 localizes to the centriole/basal body and has been implicated in endosomal trafficking, GLUT4 translocation, and insulin sensitivity, while PCNT contributes to growth, puberty, and insulin sensitivity, supporting a conserved link between centriolar proteins and endocrine function.

Monogenic insulin‐resistance syndromes have traditionally been divided into disorders of insulin signaling (including *INSR*‐ and *PIK3R1‐related* diseases) and those resulting from adipose tissue development or function (LDs) [18]. Our findings, together with evidence from *POC1A‐*, *ALMS1*‐, and *PCNT*‐associated disorders, support the emergence of a third group of conditions, centrosomal or ciliary disorders, that combine dyslipidemic insulin resistance with other systemic manifestations [11, 12, 34]. Increasing evidence indicates that the primary cilium serves as a platform for insulin receptor localization and signaling, and that disruption of this structure impairs metabolic responsiveness.

Beyond rare biallelic null variants, common *POC5* polymorphisms have been associated with complex traits such as T2D [10, 35] and adolescent idiopathic scoliosis (AIS) [7, 8, 9]. Three functional *POC5* variants (p.Ala446Thr, p.Ala455Pro, p.Ala429Val) linked to AIS affect ciliary length, cell cycle progression, and interactions with key ciliary proteins like CEP290 [7, 9], illustrating a continuum of allelic effects from mild trait associations to severe multisystem disease. Shared developmental features with *POC1A*‐related SOFT syndrome further support convergent pathogenic pathways [13].

Regarding the management of individuals carrying biallelic *POC5* variants, Vulto‐van Silfhout et al. have recommended annual cardiometabolic risk screening, as well as assessment of liver and kidney function [6]. Conversely, *POC5*, alongside other primary cilium and centrosomal components such as *POC1A*, *ALMS1*, and *PCNT*, should be systematically included in genetic testing panels for patients presenting with severe insulin resistance or lipodystrophy, particularly when associated with extra‐metabolic features such as retinal, renal, or skeletal abnormalities [14, 16, 18]. Expanding the investigation of these ciliary components in cohorts of genetically unsolved lipodystrophies will likely refine the molecular classification of these disorders and confirm the existence of a distinct class of centrosomal‐driven metabolic diseases.

Therapeutically, increased senescence raises the possibility that modulating senescence‐associated pathways could be beneficial. Although senolytic agents show promise in preclinical models of metabolic dysfunction [36], their relevance in *POC5* deficiency remains speculative given the physiological roles and heterogeneity of senescent cell populations. Beyond senescence, impaired ciliary signaling may represent an additional therapeutic axis. Notably, the transcription factor RFX3, a key regulator of primary cilium formation, is required for pancreatic beta‐cell development and function; loss of *Rfx3* leads to defective β‐cell maturation, impaired glucose‐stimulated insulin release, and glucose intolerance [37], linking ciliary transcriptional programs to endocrine regulation.

The main limitation of this study is that ex vivo cellular data were obtained from a single individual, although supported by in vitro ASC modeling. Future studies using multicenter cohorts, patient‐derived induced pluripotent stem cells, and conditional knockout animal models will be required to define the tissue‐specific roles of POC5 and evaluate therapeutic strategies. In conclusion, *POC5* deficiency emerges as a novel centrosomal ciliopathic form of LD and severe insulin resistance, redefining POC5 as a key node at the intersection of ciliary architecture and metabolic regulation, with implications for diagnosis, patient monitoring, and understanding of the mechanisms underlying systemic insulin resistance.

## Author Contributions

Conceptualization: Jérémie Gautheron and Isabelle Jéru. Methodology: Valeria Pistorio, Camille Vatier, Émilie Capel, Carine Beaupère, Martine Auclair, Virginie Steunou, Corinne Vigouroux, Jérémie Gautheron, and Isabelle Jéru. Validation and Formal analysis: Valeria Pistorio, Émilie Capel, Carine Beaupère, Martine Auclair, Jérémie Gautheron, and Isabelle Jéru. Investigation: Valeria Pistorio, Camille Vatier, Émilie Capel, Carine Beaupère, Martine Auclair, Virginie Steunou, Romain Morichon, Michael Joubert, Corinne Vigouroux, Jérémie Gautheron, Isabelle Jéru. Resources: Camille Vatier, Michael Joubert, and Corinne Vigouroux. Visualization: Valeria Pistorio, Carine Beaupère, Jérémie Gautheron, and Isabelle Jéru. Funding acquisition: Jérémie Gautheron, Camille Vatier, Corinne Vigouroux. Project administration: Jérémie Gautheron, and Isabelle Jéru. Supervision: Jérémie Gautheron, Corinne Vigouroux, and Isabelle Jéru. Writing – original draft: Valeria Pistorio, Jérémie Gautheron, and Isabelle Jéru. Writing – review and editing: Valeria Pistorio, Camille Vatier, Corinne Vigouroux, and Isabelle Jéru.

## Funding

This work was supported by the Fondation Groupama Prix de Recherche Maladies Rares 2025 (J.G.); Agence nationale de la recherche (ANR) grant ANR‐21‐CE17‐0002; Société Francophone du Diabète (SFD) grants R19114DD and C24/0316 (J.G.), as well as institutional fundings from the French Ministry of Solidarity and Health, Assistance‐Publique Hôpitaux de Paris, Sorbonne University, and by the Association Française des Lipodystrophies (AFLIP), through a donation to the Association Robert‐Debré pour la Recherche Médicale (ARDRM) (Camille Vatier, Corinne Vigouroux).

## Conflicts of Interest

The authors declare no conflicts of interest.

## Supporting information


**Video S1:** Representative U‐ExM visualization of primary cilia in wild‐type (WT) fibroblasts.

**Video S2:** Representative U‐ExM visualization of primary cilia in POC5‐deficient patient‐derived fibroblasts.

**Figure S1:** Quantification of WBs: insulin‐induced activation of signaling intermediates in fibroblasts. Quantification of Western blot experiments evaluating insulin‐induced activation of signaling intermediates in fibroblasts from controls (WT) and the patient (*POC5* ^−/−^). Representative images of the corresponding Western blots are shown in Figure 3C. Data were normalized to the insulin‐stimulated control fibroblasts of each experiment, measured in two or four independent experiments, are expressed as means ± SD. IRβ: insulin receptor β‐subunit; AKT: protein kinase B; ERK1/2: extracellular‐regulated kinase 1/2; P‐: phosphorylated proteins. Statistical analysis was performed using two‐way ANOVA followed by Bonferroni's post hoc test. ns, not significant, *p < 0.05, ***p < 0.001.
**Figure S2:** CRISPR‐Cas9‐mediated deletion of *POC5* in ASCs recapitulates impaired POC5 protein expression and defects in cilia and basal body organization. Data were obtained in ASC CRISPR‐Ctrl and CRISPR *POC5*‐KO cultured in a maintenance medium (non‐differentiated ASCs). (A) IF images of Ctrl and *POC5*‐KO cells stained against POC5 (green) and the centrosomal marker γ‐tubulin (red). Cells were counterstained with Wheat Germ Agglutinin (WGA) Alexa Fluor 633. Scale bar: 5 μm. (B) Immunocytological features of cilia and centrosome/basal body organization in ASCs CRISPR‐Ctrl and CRISPR *POC5*‐KO. Centrioles are revealed by red anti‐γ‐tubulin staining, and cilia by green anti‐ARL13B staining. Cell nuclei are stained in blue with DAPI. Representative photographs are shown, with magnification of cells depicted by rectangles. The percentage of cells with normal cilia (white boxes), abnormally shaped cilia (gray boxes), and of cells without cilia (black boxes) was evaluated on a total number of 161 CRISPR‐Ctrl and 183 CRISPR *POC5*‐KO cells and expressed as means ± SD. Ciliary phenotypes in ASC CRISPR‐control and CRISPR *POC5*‐KO cells were quantified and pooled from three independent experiments. Normal versus abnormal and absent cilia were compared using Fisher's exact test. ****p < 0.0001 vs. control.
**Figure S3:** Quantification of Western blots: insulin‐induced activation of signaling intermediates in ASCs. Quantification of Western blot experiments evaluating insulin‐induced activation of signaling intermediates in ASCs. WT: control ASCs; CRISPR‐Ctrl: ASCs transfected with a Cas9/scramble gRNA plasmid, CRISPR *POC5*‐KO: ASCs submitted to a CRISPR‐Cas9‐mediated *POC5*‐knockout. Representative images of the corresponding Western blots are shown in Figure 4C. Results are representative of four independent experiments (means ± SD). Statistical analysis was performed using two‐way ANOVA followed by Bonferroni's post hoc test. ns, not significant, *p < 0.05, **p < 0.01, ***p < 0.001.
**Figure S4:** Quantification of adipogenic factor expression and mature adipocyte markers during ASC differentiation. Quantifications of the protein expression of adipogenic factors and mature adipocyte markers, normalized to tubulin, at day 0 and day 20 of adipocyte differentiation in ASC. PPARγ, peroxisome proliferator‐activated receptor gamma; C/EBPα, CCAAT/enhancer binding protein alpha; SREBP1c, sterol regulatory element‐binding protein 1c; PLIN1, perilipin‐1; FAS, fatty acid synthase. Results are representative of three independent experiments (means ± SD). Differentiated WT ASC vs. differentiated CRISPR‐Ctrl or CRISPR *POC5*‐KO ASCs. Representative images of the corresponding Western blots are shown in Figure 5C. Statistical analysis was performed using two‐way ANOVA followed by Bonferroni's post hoc test. ns, not significant, *p < 0.05, **p < 0.01, ***p < 0.001.
**Figure S5:** Quantification of insulin‐induced activation of signaling intermediates after 20 days of ASC differentiation. Quantification of Western blot experiments evaluating insulin‐induced activation of signaling intermediates in ASCs after 20 days of adipocyte differentiation. WT: control ASCs; CRISPR‐Ctrl: ASCs transfected with a Cas9/scramble gRNA plasmid, CRISPR *POC5*‐KO: ASCs submitted to a CRISPR‐Cas9‐mediated *POC5*‐knockout. Representative images of the corresponding Western blots are shown in Figure 5D. Results are representative of three independent experiments (means ± SD). Statistical analysis was performed using two‐way ANOVA followed by Bonferroni's post hoc test. ns, not significant, *p < 0.05, **p < 0.01, ***p < 0.001.

## Data Availability

Data that support the findings of this study are included in this article or available from the corresponding authors upon reasonable request.
