# Supplementary material for: Centriolar Protein POC5 Regulates Human Adipogenesis and Cellular Senescence: Insights From a Novel Metabolic Ciliopathy
Source: FASEB J. 2026 Jul 27;40(14):e72158. doi: 10.1096/fj.202601789R (PMC13404321; doi:10.1096/fj.202601789R)
Supplement: Supplementary file 3 — Figure S1: Quantification of WBs: insulin‐induced activation of signaling intermediates in fibroblasts. Quantification of Western blot experiments evaluating insulin‐induced activation of signaling intermediates in fibroblasts from controls (WT) and the patient (POC5 −/−). Representative images of the corresponding Western blots are shown in Figure 3C. Data were normalized to the insulin‐stimulated control fibroblasts of each experiment, measured in two or four independent experiments, are expressed as means ± SD. IRβ: insulin receptor β‐subunit; AKT: protein kinase B; ERK1/2: extracellular‐regulated kinase 1/2; P‐: phosphorylated proteins. Statistical analysis was performed using two‐way ANOVA followed by Bonferroni's post hoc test. ns, not significant, *p < 0.05, ***p < 0.001. Figure S2: CRISPR‐Cas9‐mediated deletion of POC5 in ASCs recapitulates impaired POC5 protein expression and defects in cilia and basal body organization. Data were obtained in ASC CRISPR‐Ctrl and CRISPR POC5‐KO cultured in a maintenance medium (non‐differentiated ASCs). (A) IF images of Ctrl and POC5‐KO cells stained against POC5 (green) and the centrosomal marker γ‐tubulin (red). Cells were counterstained with Wheat Germ Agglutinin (WGA) Alexa Fluor 633. Scale bar: 5 μm. (B) Immunocytological features of cilia and centrosome/basal body organization in ASCs CRISPR‐Ctrl and CRISPR POC5‐KO. Centrioles are revealed by red anti‐γ‐tubulin staining, and cilia by green anti‐ARL13B staining. Cell nuclei are stained in blue with DAPI. Representative photographs are shown, with magnification of cells depicted by rectangles. The percentage of cells with normal cilia (white boxes), abnormally shaped cilia (gray boxes), and of cells without cilia (black boxes) was evaluated on a total number of 161 CRISPR‐Ctrl and 183 CRISPR POC5‐KO cells and expressed as means ± SD. Ciliary phenotypes in ASC CRISPR‐control and CRISPR POC5‐KO cells were quantified and pooled from three independent experiments. Normal ver [file FSB2-40-e72158-s002.docx]

**Supplementary Figures**

**
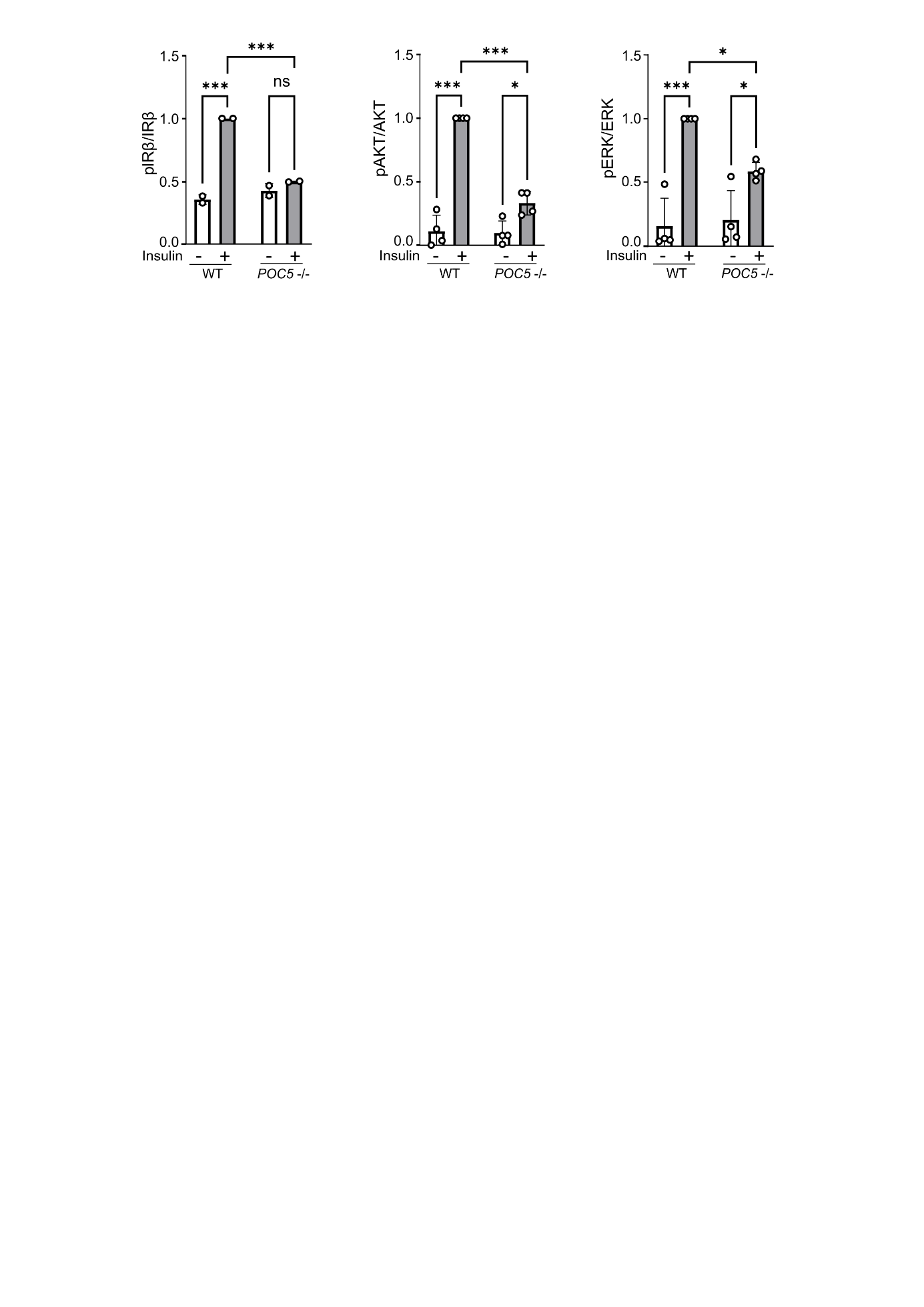
**

**Figure S1.** **Quantification of WBs: insulin-induced activation of signaling intermediates in fibroblasts.** Quantification of Western blot experiments evaluating insulin-induced activation of signaling intermediates in fibroblasts from controls (WT) and the patient (*POC5*-/-). Representative images of the corresponding Western blots are shown in Figure 3C. Data were normalized to the insulin-stimulated control fibroblasts of each experiment, measured in two or four independent experiments, are expressed as means ± SD. IRβ: insulin receptor β -subunit; AKT: protein kinase B; ERK1/2: extracellular-regulated kinase 1/2; P-: phosphorylated proteins. Statistical analysis was performed using two-way ANOVA followed by Bonferroni’s post hoc test. ns: not significant, *: p < 0.05, ***: p < 0.001.


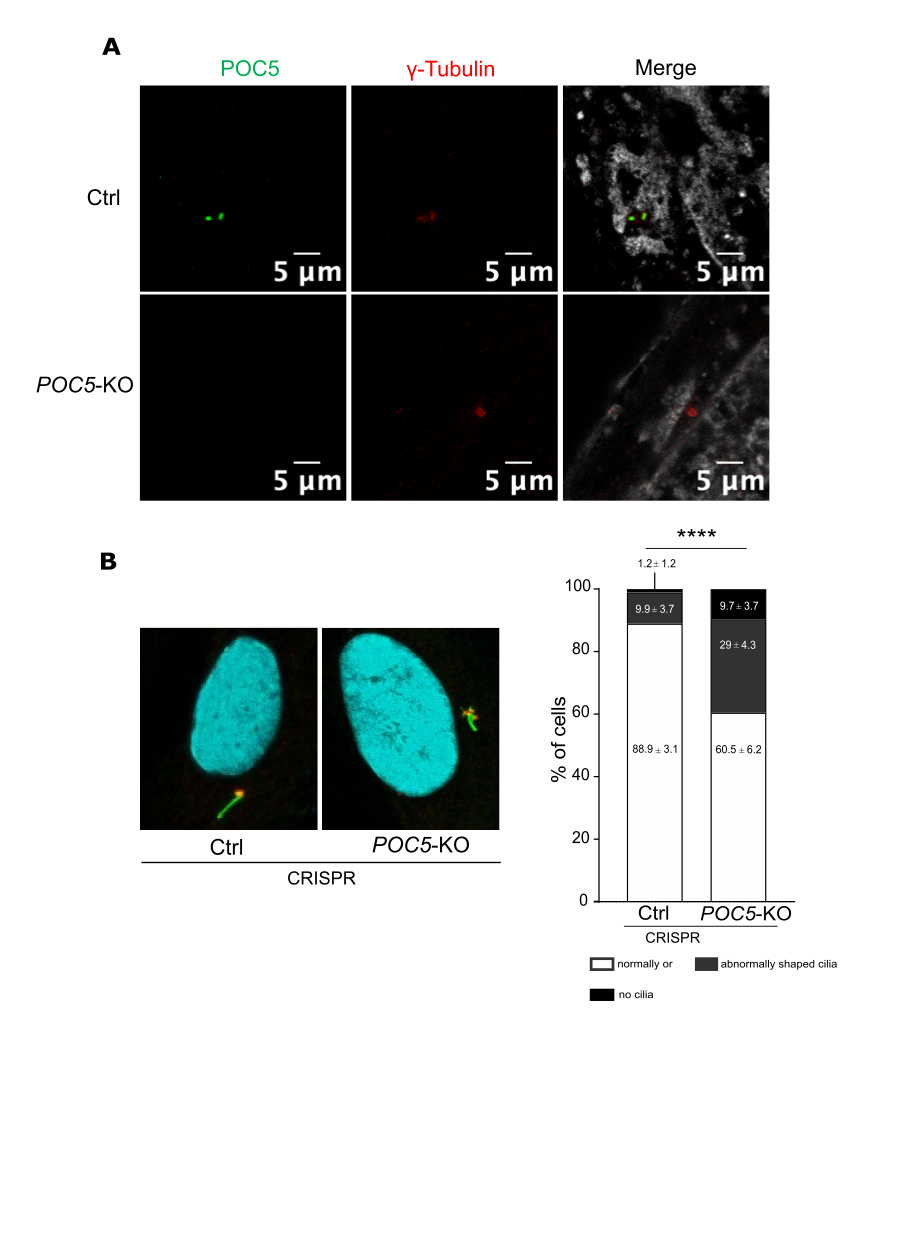


**Figure S2. CRISPR-Cas9-mediated deletion of *POC5* in ASCs recapitulates impaired POC5 protein expression and defects in cilia and basal body organization.** Data were obtained in ASC CRISPR-Ctrl and CRISPR *POC5*-KO cultured in a maintenance medium (non-differentiated ASCs). (**A**) IF images of Ctrl and *POC5*-KO cells stained against POC5 (green) and the centrosomal marker γ-tubulin (red). Cells were counterstained with Wheat Germ Agglutinin (WGA) Alexa Fluor 633. Scale bar: 5 μm. (**B**) Immunocytological features of cilia and centrosome/basal body organization in ASCs CRISPR-Ctrl and CRISPR *POC5*-KO. Centrioles are revealed by red anti-γ-tubulin staining, and cilia by green anti-ARL13B staining. Cell nuclei are stained in blue with DAPI. Representative photographs are shown, with magnification of cells depicted by rectangles. The percentage of cells with normal cilia (white boxes), abnormally shaped cilia (gray boxes), and of cells without cilia (black boxes) was evaluated on a total number of 161 CRISPR-Ctrl and 183 CRISPR *POC5*-KO cells and expressed as means ± SD. Ciliary phenotypes in ASC CRISPR-control and CRISPR *POC5*-KO cells were quantified and pooled from three independent experiments. Normal versus abnormal and absent cilia were compared using Fisher’s exact test. ****P <0.0001 vs control.

**
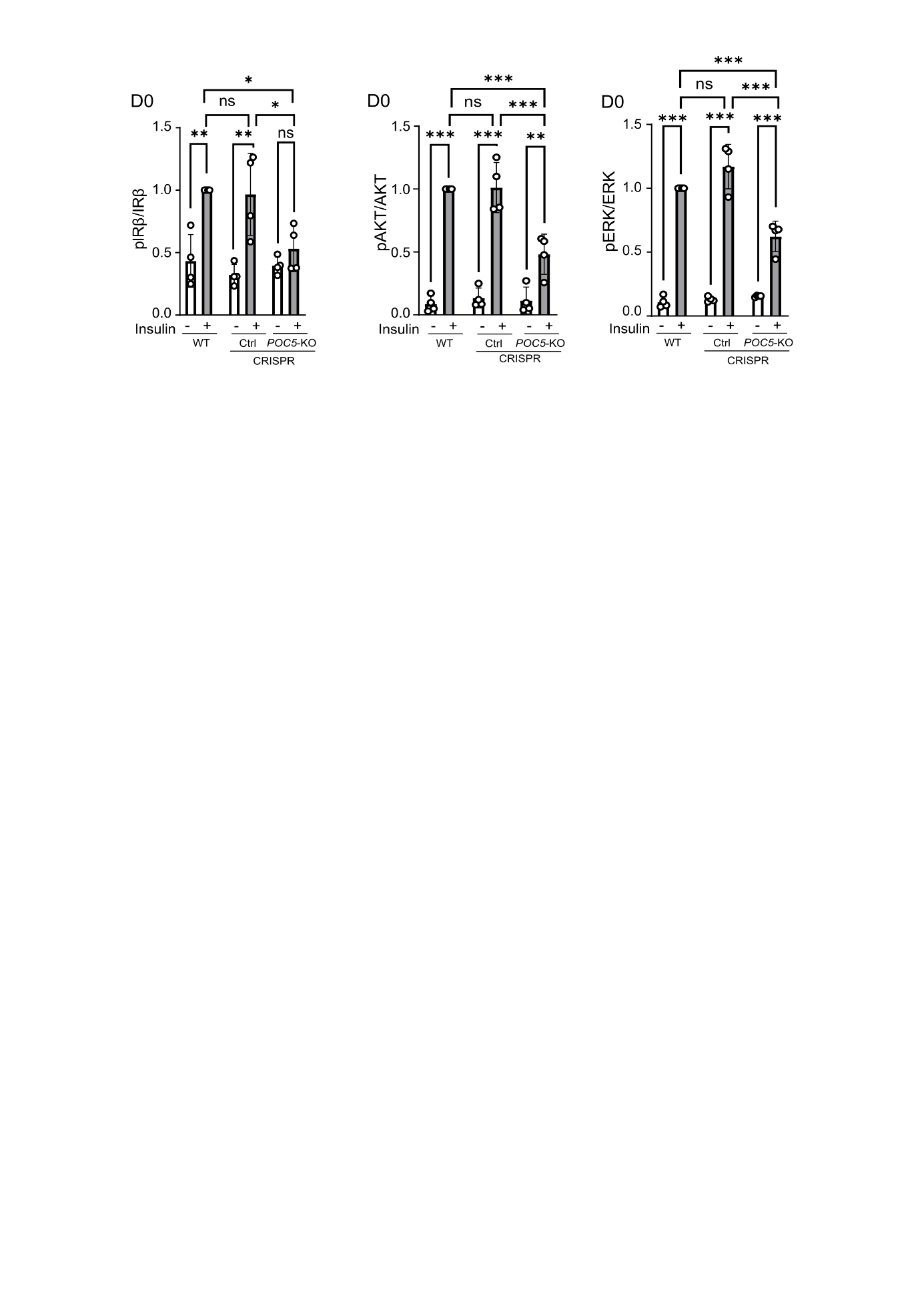
**

**Figure S3.** **Quantification of Western blots: insulin-induced activation of signaling intermediates in ASCs.** Quantification of Western blot experiments evaluating insulin-induced activation of signaling intermediates in ASCs. WT: control ASCs; CRISPR-Ctrl: ASCs transfected with a Cas9/scramble gRNA plasmid, CRISPR *POC5*-KO: ASCs submitted to a CRISPR-Cas9-mediated *POC5*-knockout. Representative images of the corresponding Western blots are shown in Figure 4C. Results are representative of four independent experiments (means ± SD). Statistical analysis was performed using two-way ANOVA followed by Bonferroni’s post hoc test. ns: not significant, *: p < 0.05, **: p < 0.01 ***: p < 0.001.

**
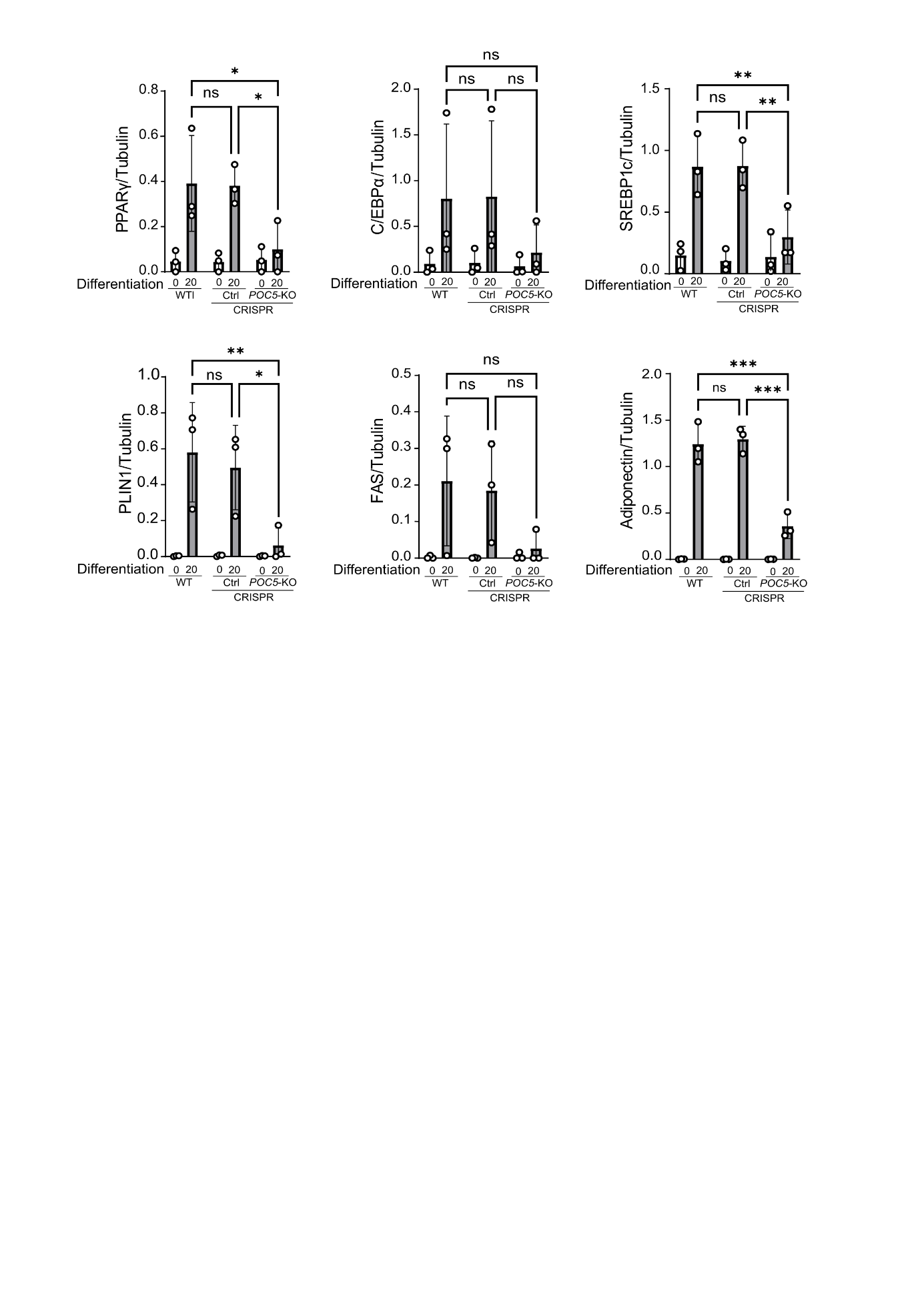
**

**Figure S4. Quantification of adipogenic factor expression and mature adipocyte markers during ASC differentiation.** Quantifications of the protein expression of adipogenic factors and mature adipocyte markers, normalized to tubulin, at day 0 and day 20 of adipocyte differentiation in ASC. PPARγ, peroxisome proliferator-activated receptor gamma; C/EBPα, CCAAT/enhancer binding protein alpha; SREBP1c, sterol regulatory element-binding protein 1c; PLIN1, perilipin-1; FAS, fatty acid synthase. Results are representative of three independent experiments (means ± SD). Differentiated WT ASC vs differentiated CRISPR-Ctrl or CRISPR *POC5*-KO ASCs. Representative images of the corresponding Western blots are shown in Figure 5C. Statistical analysis was performed using two-way ANOVA followed by Bonferroni’s post hoc test. ns: not significant, *: p < 0.05, **: p < 0.01 ***: p < 0.001.


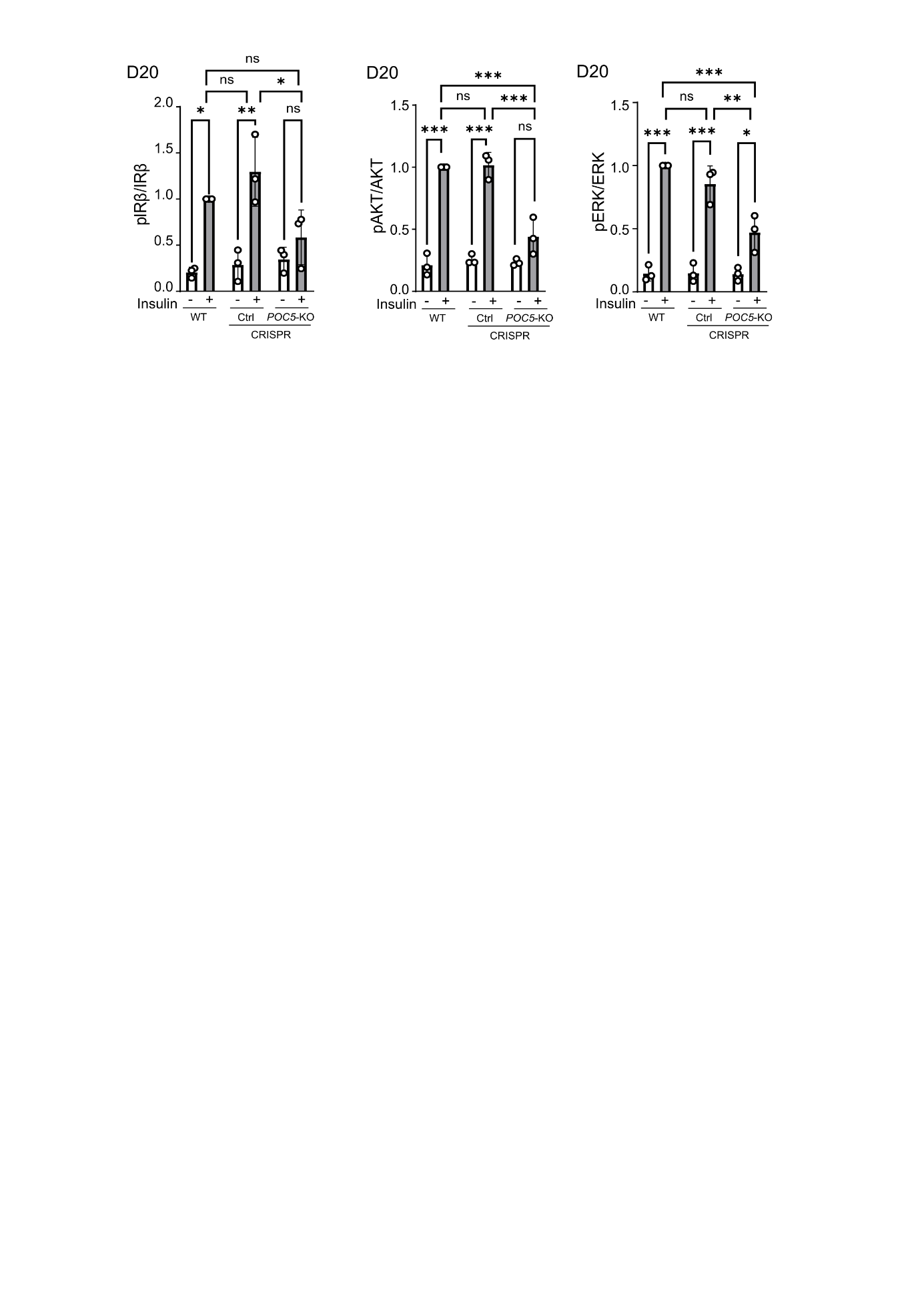


**Figure S5. Quantification of insulin-induced activation of signaling intermediates after 20 days of ASC differentiation.** Quantification of Western blot experiments evaluating insulin-induced activation of signaling intermediates in ASCs after 20 days of adipocyte differentiation. WT: control ASCs; CRISPR-Ctrl: ASCs transfected with a Cas9/scramble gRNA plasmid, CRISPR *POC5*-KO: ASCs submitted to a CRISPR-Cas9-mediated *POC5*-knockout. Representative images of the corresponding Western blots are shown in Figure 5D. Results are representative of three independent experiments (means ± SD). Statistical analysis was performed using two-way ANOVA followed by Bonferroni’s post hoc test. ns: not significant, *: p < 0.05, **: p < 0.01 ***: p < 0.001.
